# Global Prevalence of Clinically Significant Insomnia Among Cancer Patients: A Systematic Review and Meta‐Analysis

**DOI:** 10.1002/brb3.71857

**Published:** 2026-11-13

**Authors:** Ting Du, Linyu Luo, Langli Gao, Juan Lv

**Affiliations:** ^1^ Center of Gerontology and Geriatrics, West China Hospital Sichuan University Chengdu Sichuan China

**Keywords:** cancer, clinically significant insomnia, epidemiology, meta‐analysis, prevalence

## Abstract

**Background**: Clinically significant insomnia is associated with multiple adverse health outcomes in cancer patients. This study aimed to examine the global prevalence of clinically significant insomnia among cancer patients and to determine the moderators of the prevalence estimate.

**Methods**: Literature search was conducted from inception to October 1, 2025, across 11 major electronic databases, including PubMed, Web of Science, Scopus, Embase, PsycINFO, Cochrane Library, CINAHL, CNKI, Wanfang, VIP, and CBM. Methodological quality was evaluated using the Joanna Briggs Institute (JBI) critical appraisal tool. A random‐effects model was employed to calculate the pooled prevalence using Stata 15.0. Using the *I* ^2^ statistic to assess heterogeneity, we also conducted subgroup analyses and meta‐regression to identify its possible sources.

**Results**: On the basis of a total of 39 studies comprising 14,094 cancer patients, the pooled prevalence of clinically significant insomnia was 34.6% (95% CI: 27.2–44.1). A total of 25 studies were assessed as having a low overall risk of bias and 14 as having a moderate risk. Subgroup and meta‐regression analyses identified country income level and sample size as significant determinants of the pooled prevalence.

**Conclusions**: Our findings indicated a high global prevalence of clinically significant insomnia in cancer patients, highlighting the urgency to integrate sleep health management into routine oncology care to optimize their health outcomes.

## Graphical abstract

This is a meta‐analysis of 39 studies comprising 14,094 cancer patients across 16 countries. The pooled global prevalence was 34.6% (95% CI: 27.2–44.1). Subgroup analyses revealed a higher prevalence in high‐income countries compared to low‐income countries. Meta‐regression showed that smaller sample size was associated with higher prevalence estimates. The conclusion emphasizes that approximately one in three cancer patients suffers from clinically significant insomnia, underscoring the urgent need to integrate routine sleep health screening and evidence‐based interventions into standard oncology care.

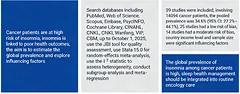

## 1 Introduction

As a persistent public health threat, cancer affects millions across the globe (Sun et al. 2025). With the global cancer burden constantly increasing, the focus of cancer supportive care is gradually turning from pursuing prolonged survival to comprehensively improving the quality of life of cancer patients (Belcher et al. 2023). Sleep disorders, particularly clinically significant insomnia, have emerged as a common yet under‐recognized health issue distressing cancer patients and diminishing their quality of life (Aggeli et al. 2021; Han et al. 2025).

Insomnia symptoms are prevalent among cancer patients, but not all insomnia problems constitute clinically significant disorders (Cha et al. 2017). Clinically significant insomnia is characterized not only by difficulties initiating or maintaining sleep, or early awakening, but also by severe impairment in daytime functioning, such as marked fatigue, irritability, cognitive decline, or diminished social functioning (Cho et al. 2023; Desai et al. 2013). These features distinguish it from mild sleep disturbances, highlighting its clinical significance as a distinct disease rather than merely a symptom accompanying cancer (Hoang et al. 2020). Furthermore, current evidence indicates that clinically significant insomnia is intimately associated with a variety of adverse outcomes among cancer patients (Casault et al. 2012; Liou et al. 2019; Ma et al. 2020). Specifically, it has been correlated with cancer‐related fatigue, impaired cognitive function, increased reliance on analgesic medications, and potentially diminished survival (Casault et al. 2012; Liou et al. 2019; Ma et al. 2020). Therefore, these findings highlighted the clinical relevance of screening and administration for clinically significant insomnia in cancer patients.

The concern over clinically significant insomnia in cancer patients has been growing. To date, numerous studies worldwide have investigated this condition, yet reported prevalence estimates exhibit substantial heterogeneity, with rates varying considerably from 7.9% to 79.6% across different populations and settings (Desautels et al. 2017; Fleming et al. 2019; Madkhali et al. 2024; Mercadante et al. 2015). These discrepancies may arise from cancer type and stage, chemotherapy, analgesic use, and the various measurement tools or cutoff values employed. In addition, the prevention and treatment of clinically significant insomnia would be directed towards those who screen positive. However, no meta‐analysis has reported the pooled global prevalence of clinically significant insomnia in cancer patients. Therefore, this study aimed to examine the global prevalence of clinically significant insomnia among cancer patients and to explore the influence of various study characteristics on the prevalence estimate.

## 2 Methods

This study adhered to the Preferred Reporting Items for Systematic reviews and Meta‐Analyses (PRISMA) guidelines (Page et al. 2021). The protocol was registered with PROSPERO (CRD420251169857).

### 2.1 Search Strategy

Literature search was conducted in 11 electronic databases, including PubMed, Web of Science, Scopus, Embase, PsycINFO, Cochrane Library, CINAHL, China Knowledge Resource Integrated Database (CNKI), Wanfang Database, Weipu Database (VIP), and Chinese Biomedical Database (CBM), for relevant articles published from their inception until October 1, 2025, with language restricted to English and Chinese. The search strategy employed MeSH and other keywords, including “neoplasms,” “cancer,” “insomnia,” “sleep disturbances,” “sleep disorders,” “sleep initiation and maintenance disorders,” and “prevalence.” The specific syntax is detailed in Table S1. Additionally, the reference lists of the included studies and other literature reviews were manually screened to identify potential publications.

### 2.2 Eligibility Criteria

Studies were included if the following criteria were met: (1) Participants were adult cancer patients (aged ≥18 years); (2) with a cross‐sectional or cohort design; (3) clinically significant insomnia was determined using validated self‐reported instruments; (4) the prevalence of clinically significant insomnia was reported, either as percentages or as raw numbers that allowed for the calculation of percentages; and (5) in case of duplicate publications, the largest sample study was retained. The exclusion criteria (1) reviews, case reports, comments, editorials, or conference abstracts; (2) not published in English or Chinese; and (3) unavailable in full text.

### 2.3 Data Extraction

A structured Excel form was used for data extraction. Two reviewers independently extracted from each study the first author, publication year, country, study design, sample size, mean age, cancer category, proportion of females, assessment tool, and prevalence of clinically significant insomnia. In addition, the extracted data were checked to verify and resolve any discrepancies.

### 2.4 Quality Assessment

Two reviewers independently assessed the risk of bias using the Joanna Briggs Institute (JBI) critical appraisal tool for prevalence studies (Munn et al. 2015). The tool consists of nine items, each requiring a determination of “yes,” “no,” or “unclear/not applicable.” Studies were ultimately classified into three categories based on the percentage of entries rated “yes”: low risk (≥70%), moderate risk (50%–69%), or high risk (≤49%). Any disagreements were resolved by consulting a third reviewer. A final consensus was then determined through collective discussion.

### 2.5 Data Analysis

Statistical analyses were performed with Stata 15.0. A random‐effects model was employed to calculate the prevalence of clinically significant insomnia in cancer patients, along with the corresponding 95% confidence intervals (CIs). Heterogeneity across studies was assessed using the *I* ^2^ statistic, with an *I* ^2^ value ≥50% indicating substantial heterogeneity (Higgins et al. 2003). Subgroup analyses were conducted to explore potential sources of heterogeneity on the basis of study design, country income level, cancer category, and the assessment tool used. Additionally, we conducted univariate meta‐regression analyses to explore moderators of prevalence, adjusting for publication year, sample size, and proportion of females. Publication bias was evaluated using a funnel plot for visual inspection and Egger's test for statistical evaluation. A leave‐one‐out sensitivity analysis was carried out to evaluate the robustness of the findings. A two‐sided *p* value < 0.05 was considered statistically significant.

## 3 Results

### 3.1 Literature Selection

The initial database search identified 11,530 publications, and five additional records were selected from the reference lists. After eliminating duplicates, 6235 records underwent title and abstract screening, with 232 proceeding to full‐text review. Finally, 39 studies that met the inclusion criteria were included in this study. The selection process is shown in Figure 1.

**FIGURE 1:**
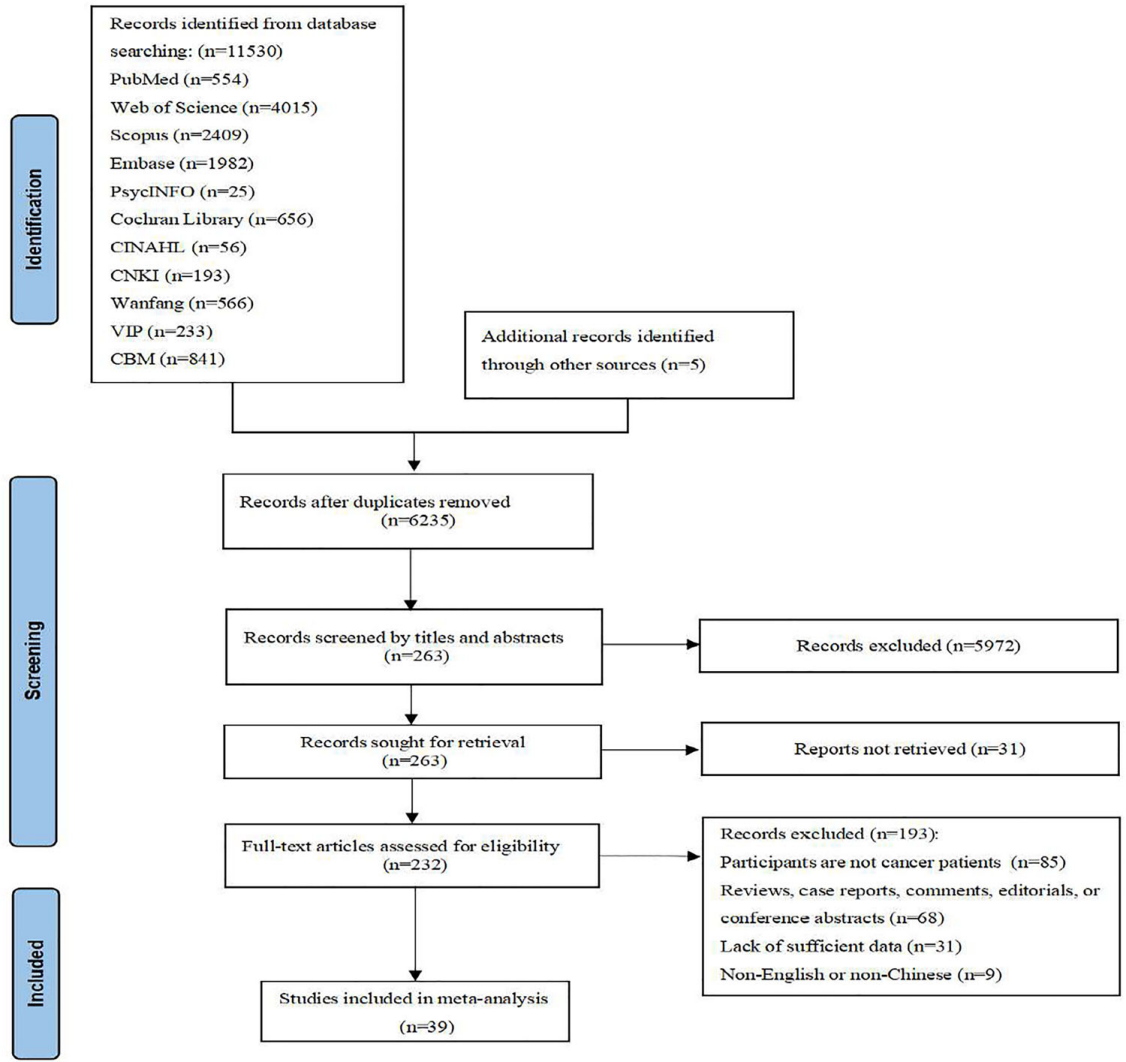
Flow diagram of study selection.

### 3.2 Study Characteristics

A total of 39 studies (Aggeli et al. 2021; Cha et al. 2017; Chen et al. 2021; Cho et al. 2023; Desai et al. 2013; Desautels et al. 2017; Dirksen et al. 2009; Eslamijouybari et al. 2025; Fleming et al. 2019; Galvin et al. 2022; Geng et al. 2023; Hajj et al. 2021; Haque et al. 2021; He 2022; Hinz et al. 2025; Hoang et al. 2020; Huang et al. 2024; Juanjuan et al. 2020; Li et al. 2024; Liao and Zhang 2023; Liou et al. 2019; Liu et al. 2023; Madkhali et al. 2024; Mercadante et al. 2015; Nishiura et al. 2015; Noyan et al. 2023; Pang et al. 2022; Price et al. 2009; Ruiz‐Marin et al. 2021; Savard et al. 2005; Sun et al. 2011, 2020; Taskaynatan et al. 2025; Trendowski et al. 2025; Ueno et al. 2022; Wang et al. 2022; Yeo et al. 2021; Yi et al. 2022; Zhang et al. 2024) examining the prevalence of clinically significant insomnia in cancer patients were included. These studies were conducted across 16 countries, including China (*n* = 13), the United States (*n* = 5), Korea (*n* = 4), Turkey (*n* = 3), Canada (*n* = 2), Japan (*n* = 2), Greece (*n* = 1), the United Kingdom (*n* = 1), New Zealand (*n* = 1), Lebanon (*n* = 1), Germany (*n* = 1), Vietnam (*n* = 1), Saudi Arabia (*n* = 1), Italy (*n* = 1), Australia (*n* = 1), and Spain (*n* = 1). The study designs were mainly cross‐sectional (*n* = 32), with a minority of cohort studies (*n* = 7). In addition, the majority of studies explored solid tumors, with breast cancer accounting for the largest proportion (*n* = 14). Clinically significant insomnia was assessed using the Insomnia Severity Index (ISI) (*n* = 28) and the Athens Insomnia Scale (AIS) (*n* = 11), with the reported prevalence ranging from 7.9% to 79.6%. Characteristics of the included studies are summarized in Table 1.

**TABLE 1:** Characteristics of included studies

Study	Country	Study design	Sample size	Mean age (years)	Cancer category	Female (%)	Assessment tool	Prevalence (%)
Aggeli et al. (2021)	Greece	Cross‐sectional	170	NR	Solid (breast cancer)	100	AIS	53.5
Cha et al. (2017)	Korea	Cross‐sectional	208	50.72 ± 11.49	Multiple cancers	79.3	ISI	45.7
Chen et al. (2021)	China	Cross‐sectional	834	NR	Solid (breast cancer)	100	ISI	14.7
Cho et al. (2023)	Korea	Cross‐sectional	146	56.7 ± 11.6	Multiple cancers	74	ISI	74.7
Desai et al. (2013)	The United States	Cross‐sectional	413	NR	Solid (breast cancer)	100	ISI	18.3
Desautels et al. (2017)	Canada	Cohort	962	57	Multiple cancers	64.4	ISI	7.9
Dirksen et al. (2009)	The United States	Cross‐sectional	51	74	Solid (prostate cancer)	0	ISI	52.9
Eslamijouybari et al. (2025)	Iran	Cross‐sectional	468	45.03 ± 10.57	Solid (breast cancer)	100	ISI	19.4
Fleming et al. (2019)	The United Kingdom	Cohort	173	58	Solid (breast cancer)	100	ISI	8.1
Galvin et al. (2022)	New Zealand	Cross‐sectional	142	NR	Solid (prostate cancer)	0	ISI	21.1
Geng et al. (2023)	China	Cohort	47	NR	Solid (breast cancer)	100	AIS	74.5
Hajj et al. (2021)	Lebanon	Cross‐sectional	112	NR	Solid (breast cancer)	100	ISI	27.7
Haque et al. (2021)	The United States	Cohort	315	70.8 ± 6.4	Solid (breast cancer)	100	ISI	8.3
He (2022)	China	Cross‐sectional	120	53.2	Solid (breast cancer)	100	AIS	13.3
Hinz et al. (2025)	Germany	Cross‐sectional	533	52.3 ± 12.5	Solid (breast cancer)	100	ISI	51.3
Hoang et al. (2020)	Vietnam	Cross‐sectional	213	53.1 ± 11.3	Multiple cancers	60	ISI	42.8
Huang et al. (2024)	China	Cross‐sectional	281	NR	Multiple cancers	37.4	ISI	25.9
Juanjuan et al. (2020)	China	Cross‐sectional	658	NR	Solid (breast cancer)	100	ISI	16.9
Li et al. (2024)	China	Cross‐sectional	372	59.12 ± 10.37	Solid (lung cancer)	36.3	AIS	37.4
Liao and Zhang (2023)	China	Cross‐sectional	100	59.00 ± 10.43	Multiple cancers	41	AIS	41
Liou et al. (2019)	The United States	Cross‐sectional	1072	62.1 ± 9.9	Solid (lung cancer)	100	ISI	19.9
Liu et al. (2023)	China	Cross‐sectional	176	66	Solid (non‐melanoma skin cancer)	52.8	AIS	26.7
Madkhali et al. (2024)	Saudi Arabia	Cross‐sectional	291	NR	Multiple cancers	60.5	ISI	68
Mercadante et al. (2015)	Italy	Cross‐sectional	820	69.7 ± 12.7	Multiple cancers	47.7	AIS	79.6
Nishiura et al. (2015)	Japan	Cross‐sectional	50	71.8 ± 3.5	Solid (lung cancer)	30	AIS	32
Noyan et al. (2023)	Turkey	Cross‐sectional	126	42.08	Multiple cancers	46.8	ISI	14.3
Pang et al. (2022)	China	Cross‐sectional	696	NR	Multiple cancers	29.6	ISI	8.6
Price et al. (2009)	Australia	Cohort	772	NR	Solid (ovarian cancer)	100	ISI	16.8
Ruiz‐Marin et al. (2021)	Spain	Cross‐sectional	130	57.9 ± 12.4	Multiple cancers	52.3	ISI	24.6
Savard et al. (2005)	Canada	Cross‐sectional	327	66 ± 6.7	Solid (prostate cancer)	0	ISI	18
Sun et al. (2011)	China	Cross‐sectional	195	56.74 ± 13.31	Multiple cancers	59.5	AIS	22.6
Sun et al. (2020)	China	Cross‐sectional	434	62.57 ± 10.98	Solid (colorectal cancer)	41.5	AIS	38.2
Taskaynatan et al. (2025)	Turkey	Cross‐sectional	206	56.1 ± 11.7	Multiple cancers	100	ISI	17
Trendowski et al. (2025)	The United States	Cohort	718	61.3 ± 11.4	Multiple cancers	51.7	ISI	11.3
Ueno et al. (2022)	Japan	Cross‐sectional	759	59 ± 12	Solid (breast cancer)	100	AIS	37.4
Wang et al. (2022)	China	Cohort	101	48.6	Solid (head and neck cancer)	23.8	ISI	61.4
Yeo et al. (2021)	Korea	Cross‐sectional	159	54.84 ± 11.55	Multiple cancers	81.8	ISI	57.2
Yi et al. (2022)	Korea	Cross‐sectional	235	56.1 ± 11.2	Multiple cancers	74.5	ISI	65.1
Zhang et al. (2024)	China	Cross‐sectional	509	50.34 ± 10.0	Solid (breast cancer)	100	ISI	12.4

Abbreviations: AIS, Athens Insomnia Scale; ISI, Insomnia Severity Index.

### 3.3 Quality Assessment Results

The quality assessment results are outlined in Table S2. Overall, 25 (64.1%) studies showed low risk and 14 (35.9%) exhibited moderate risk.

### 3.4 Prevalence of Clinically Significant Insomnia Among Cancer Patients

Substantial heterogeneity was observed across studies (*I* ^2^ = 99.1%), prompting the use of a random‐effects model. The pooled global prevalence of clinically significant insomnia in cancer patients was 34.6% (95% CI: 28.0–41.3; *p* < 0.001), as presented in Figure 2.

**FIGURE 2:**
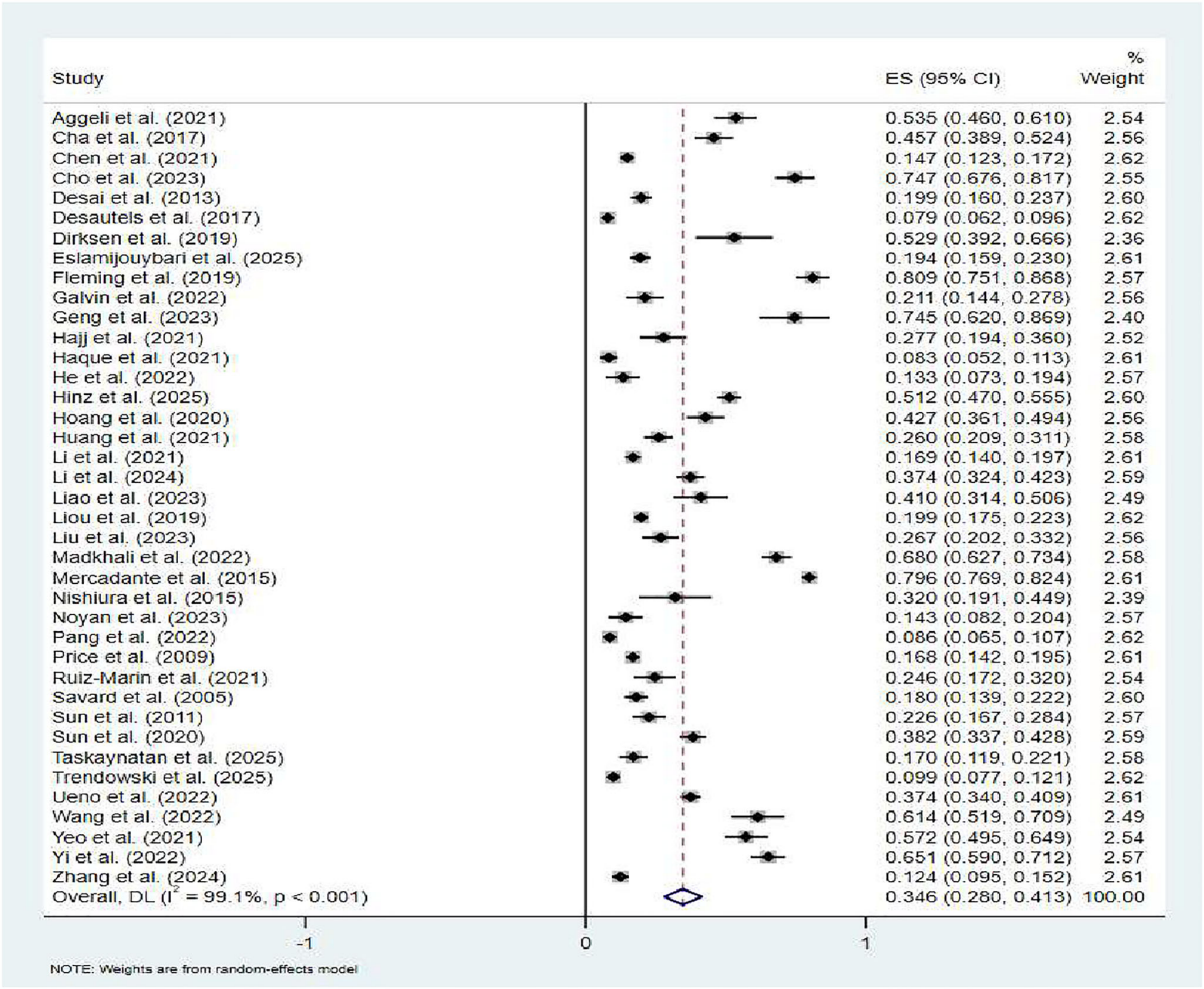
The pooled prevalence of clinically significant insomnia among cancer patients.

### 3.5 Subgroup Analysis and Meta‐Regression Analysis

Subgroup analysis was conducted to investigate heterogeneity across studies (Table 2). When stratified by country income level (Figure S1), the estimated prevalence of clinically significant insomnia among cancer patients was significantly higher in high‐income countries (41.6%, 95% CI: 30.8–52.4) than in middle‐income countries (25.4%, 95% CI: 20.2–30.6). As shown in Figure S2, cross‐sectional (34.3%, 95% CI: 26.8–41.8) and cohort studies (36.1%, 95% CI: 22.7–49.6) had comparable pooled prevalence of clinically significant insomnia in cancer patients. The subgroup analysis by cancer category (Figure S3) revealed that multiple cancers (37.8%, 95% CI: 24.0–51.5) had the highest prevalence of clinically significant insomnia, whereas prostate cancer had the lowest (28.9%, 95% CI: 14.8–43.0). When analyzed using various assessment tools (Figure S4), the estimated prevalence of clinically significant insomnia for AIS and ISI was 41.4% (95% CI: 26.6–56.2) and 31.9% (95% CI: 25.7–38.1), respectively.

**TABLE 2:** Subgroup analyses of the prevalence of clinically significant insomnia among cancer patients.

Subgroups	Number of included studies	Prevalence (%)	95% CI	*p* value
Country income level				0.008
High income	22	41.6	30.8–52.4	
Middle income	17	25.4	20.2–30.6	
Study design				0.813
Cross‐sectional	32	34.3	26.8–44.5	
Cohort	7	36.1	22.7–49.6	
Cancer category				0.892
Breast cancer	13	32.6	22.9–42.3	
Multiple cancers	16	37.8	24.0–51.5	
Prostate cancer	3	28.9	14.8–43.0	
Lung cancer	3	29.5	15.7–43.2	
Other cancers	4	35.4	19.2–51.5	
Assessment tool				0.245
AIS	11	41.4	26.6–56.2	
ISI	28	31.9	25.7–38.1	

Abbreviations: AIS, Athens Insomnia Scale; ISI, Insomnia Severity Index.

Univariate meta‐regression analysis identified a significant association between sample size and the pooled prevalence of clinically significant insomnia among cancer patients (*β* = −0.0002653, *p* = 0.035), accounting for 9.05% of the between‐study variance. However, no significant correlation was detected with publication year, mean age, or the proportion of female participants (Table S3).

### 3.6 Publication Bias and Sensitivity Analysis

No evidence of publication bias was observed, as indicated by a symmetric funnel plot (Figure 3) and a nonsignificant Egger's test (*t* = 0.02, *p* = 0.982; Figure S5). A leave‐one‐out sensitivity analysis further confirmed this finding, indicating that no individual study substantially affected the overall prevalence estimate (Table S4). These results validated the stability of the primary meta‐analysis findings.

**FIGURE 3:**
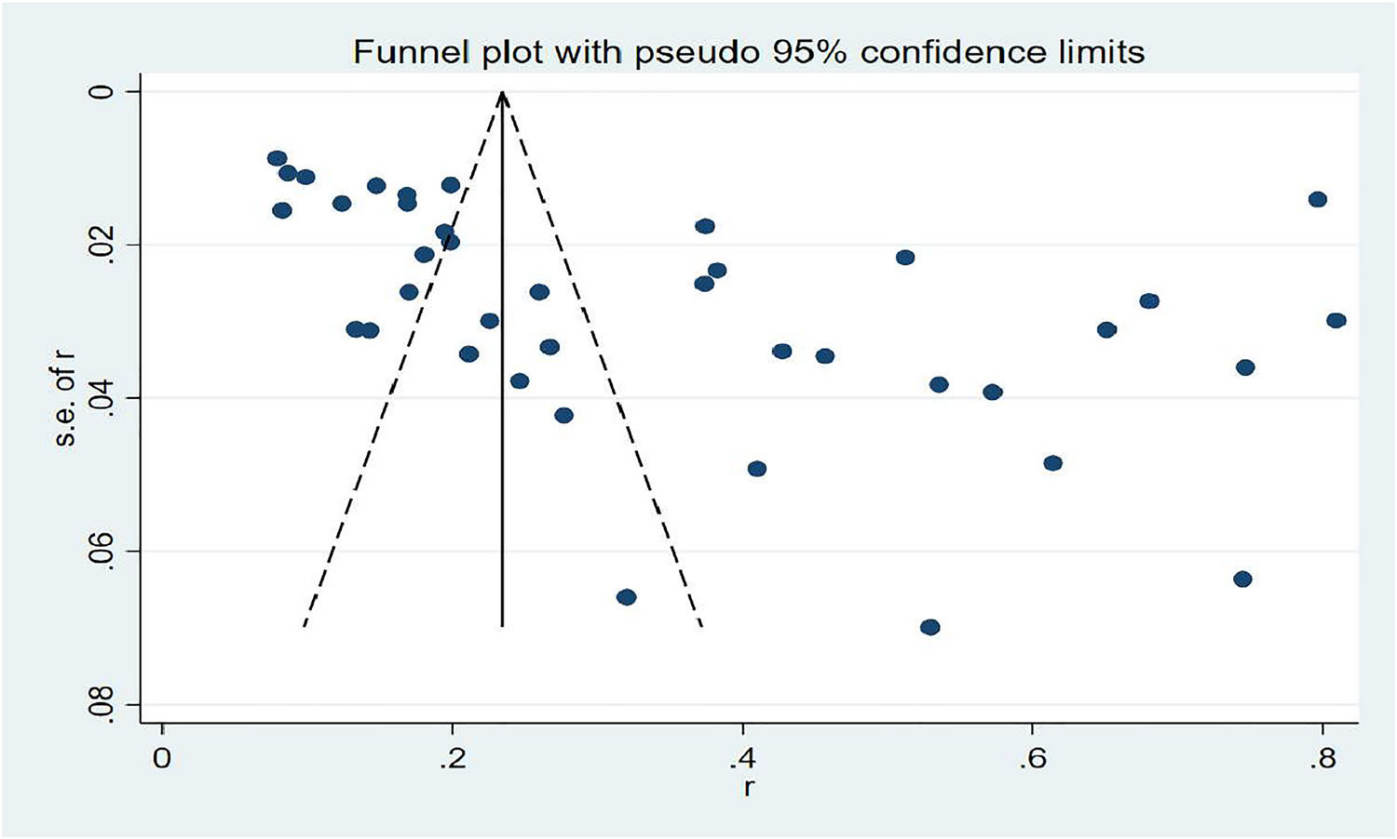
Funnel plot of prevalence of clinically significant insomnia in cancer patients.

## 4 Discussion

Given the distress and quality of life impairment caused by clinically significant insomnia in cancer patients (Wang et al. 2022; Ueno et al. 2022), this study measured its point prevalence worldwide in 14,094 participants across 17 countries. Our pooled global prevalence was 34.6%, meaning that approximately one in three cancer patients suffers from clinically significant insomnia. This rate is significantly higher than that in the general adult population, confirming that cancer confers a markedly increased risk for severe sleep disturbance. The magnitude of this burden is comparable to that of other common cancer‐related symptoms, such as fatigue and pain, yet insomnia remains underrecognized in routine oncology practice. The elevated prevalence observed in our study is not surprising given the multifactorial nature of sleep disruption in the context of cancer. Cancer patients are exposed to a complex interplay of physical, psychological, and treatment‐related factors that collectively disturb normal sleep architecture. For example, cancer‐related pain and fatigue are consistently associated with insomnia severity (Sun et al. 2020; Taskaynatan et al. 2025). Psychological distress, including anxiety and depression, further perpetuates sleep difficulties through hyperarousal and maladaptive cognitions (Sun et al. 2020; Taskaynatan et al. 2025). In addition, active treatments, such as chemotherapy, radiotherapy, endocrine therapy, and targeted agents, can directly induce insomnia through neuroendocrine alterations or circadian rhythm disruption (Desai et al. 2013; Hoang et al. 2020). Hospitalization, frequent medical visits, and polypharmacy also contribute to fragmented sleep. The cumulative effect of these factors may explain why the prevalence of clinically significant insomnia in cancer patients far exceeds that in the general population. Given that clinically significant insomnia is associated with reduced quality of life, increased fatigue, cognitive dysfunction, and potentially poorer cancer outcomes (Liou et al. 2019; Ma et al. 2020), systematic screening should become a routine component of cancer supportive care. From a pathophysiological perspective, insomnia in cancer patients may have consequences beyond symptom burden. Sleep disturbance has been linked to alterations in brain networks, including changes in resting‐state functional connectivity within subcortical and default mode networks that are associated with cognitive dysfunction and hyperarousal (Phillips et al. 2022). Preclinical and clinical evidence further suggests that sleep deprivation activates neuroinflammatory pathways, leading to increased pro‐inflammatory cytokines and cognitive impairment (Antonioni 2026; Winkler et al. 2023). These mechanisms may intersect with cancer pathophysiology and treatment response, underscoring the importance of recognizing and treating insomnia as part of comprehensive oncology care (Salvalaggio et al. 2024). For patients who screen positive, evidence‐based interventions, such as cognitive behavioral therapy for insomnia, should be made accessible to reduce prevalence and improve quality of life (Belcher et al. 2023; Savard et al. 2005). In addition, pharmacotherapy, including dual orexin receptor antagonists and benzodiazepines, may also be a possible and potentially useful option for selected patients (Bottignole et al. 2026; Rollo, Antonioni, and Di Lorenzo 2026, Rollo, Antonioni, Manzoli, et al. 2026).

Subgroup analysis revealed a markedly higher pooled prevalence of clinically significant insomnia in cancer patients in high‐income countries (41.6%) compared with low‐income countries (25.4%). The observed difference may be attributed to several factors. First, greater clinical awareness and more standardized application of diagnostic criteria for insomnia in high‐income countries likely lead to higher detection rates (Desautels et al. 2017). As a result, clinically significant insomnia is more likely to be identified and recorded in these regional healthcare systems. By contrast, insomnia is potentially underreported in low‐income countries, where clinical attention and resources are focused on more immediate and life‐threatening complications of cancer (Hoang et al. 2020). Second, patients in high‐income countries often have longer survival due to better access to advanced treatments, which increases the window of opportunity for insomnia to develop and be documented. Third, cultural differences in symptom reporting and help‐seeking behavior may also contribute; patients in some low‐income settings may normalize sleep disturbances as an inevitable part of cancer. Furthermore, the included studies were predominantly conducted in high‐income countries, which may have led to increased detection of positive cases. These findings highlight the urgent need to incorporate sleep health into comprehensive cancer care strategies, particularly in underserved areas.

Meta‐regression analysis revealed that sample size is a significant contributor to the prevalence of clinically significant insomnia among cancer patients, demonstrating that studies with smaller sample sizes may report higher prevalence estimates. Small‐sample research tends to concentrate on specific clinical subgroups with more severe conditions or exhibits poor methodological stability, making it more vulnerable to extreme variations in estimated values (Mercadante et al. 2015). Moreover, no meaningful association was detected between publication year, mean age, and proportion of female patients, and the prevalence of clinically significant insomnia among cancer patients. These results suggested that the pooled prevalence estimates in this study did not undergo significant changes over time and remained stable across participants with diverse age and gender compositions. Notably, the absence of an age or gender effect contrasts with findings from the general population, where female gender and older age are consistently associated with higher insomnia risk. This discrepancy implies that the cancer context may override typical demographic differences, possibly because cancer‐related stressors and treatments exert a uniformly potent effect on sleep across all patient subgroups. Last but not least, although sample size explained a part of the heterogeneity across included studies, most remained unexplained, likely owing to unmeasured clinical factors, such as cancer stage, treatment types, comorbidities, and socioeconomic status. The inconsistent reporting of these variables across studies highlights a critical need for standardized data collection in future supportive care research.

## 5 Limitation

Several limitations should be considered. First, substantial heterogeneity was detected among the pooled prevalence of clinically significant insomnia in cancer patients due to data derived from various study designs, assessment tools, and geographic locations, which remains largely unexplained by the variables examined. Second, the comprehensiveness of the prevalence estimates may be limited by the inclusion of only English and Chinese studies. Third, the included studies did not distinguish between insomnia reactive to the cancer context and pre‐existing chronic insomnia, as the ISI and AIS assess current symptom severity without capturing insomnia duration or onset relative to cancer diagnosis. Therefore, the pooled prevalence should be interpreted as the prevalence of clinically significant insomnia symptoms among cancer patients rather than the prevalence of chronic insomnia disorder. Fourth, we restricted inclusion to cohort and cross‐sectional studies because prevalence estimation requires cases and denominators from the same defined population. Case–control and trial data are selected samples and may not be representative. This may have excluded some relevant data, but the criterion was prespecified to preserve validity. Fifth, our inclusion criteria did not restrict treatment status, and most included studies did not report whether participants were receiving insomnia treatment. This may have influenced the observed prevalence, as treated patients may have had better controlled symptoms, and differences in treatment status may have contributed to the heterogeneity. Finally, excluding studies that relied on non‐specific screening scales may have underestimated the prevalence of clinically significant insomnia in cancer patients.

## 6 Conclusion

In conclusion, this meta‐analysis revealed a high prevalence of clinically significant insomnia in cancer patients, with the estimate being significantly affected by country income level and sample size. These findings highlight the necessity of prioritizing sleep health among cancer patients. Targeted educational interventions are pivotal to alleviating the prevalence of clinically significant insomnia and to fostering health outcomes in this vulnerable group.

## Author Contributions

**Ting Du**: conceptualization, methodology, data curation, formal analysis, writing – original draft. **Linyu Luo**: data curation, formal analysis, methodology. **Langli Gao**: conceptualization, methodology, supervision, writing – review and editing. **Juan Lv**: conceptualization, methodology, writing – review and editing, supervision.

## Funding

The authors have nothing to report.

## Ethics Statement

This work was exempt from ethical approval on account of being a systematic review.

## Conflicts of Interest

The authors declare no conflicts of interest.

## Supporting information




**Supplementary Material**: brb371857‐sup‐0001‐SuppMat.docx

## Data Availability

The corresponding author will make data available upon request.
