# Supplementary material for: Global Prevalence of Clinically Significant Insomnia Among Cancer Patients: A Systematic Review and Meta‐Analysis
Source: Brain Behav. 2026 Nov 13;16(11):e71857. doi: 10.1002/brb3.71857 (PMC13768738; doi:10.1002/brb3.71857)
Supplement: Supplementary file 1 [file BRB3-16-e71857-s001.docx]

**Supplemental Materials**

Table S1. The search strategies.

| **Databases** | **Step** | **Search Strategies** |
| --- | --- | --- |
| PubMed | #1 | "[neoplasms](https://www.ncbi.nlm.nih.gov/mesh/68009369)"[Mesh Terms] Sort by: Most Recent |
|  | #2 | "cancer"[Title/Abstract] OR "tumor"[Title/Abstract] OR "tumour"[Title/Abstract] OR "carcinoma"[Title/Abstract] OR "malignancy"[Title/Abstract] Sort by: Most Recent |
|  | #3 | "sleep"[Mesh Terms] Sort by: Most Recent |
|  | #4 | "[sleep initiation and maintenance disorders](https://www.ncbi.nlm.nih.gov/mesh/68007319)"[Mesh Terms] Sort by: Most Recent |
|  | #5 | "insomnia"[Title/Abstract] OR "sleep"[Title/Abstract] OR "sleep disturbances"[Title/Abstract] OR "sleep disorders"[Title/Abstract] "[sleep initiation and maintenance disorders](https://www.ncbi.nlm.nih.gov/mesh/68007319)"[Title/Abstract] Sort by: Most Recent |
|  | #6 | "prevalence"[Mesh Terms] Sort by: Most Recent |
|  | #7 | "incidence"[Mesh Terms] Sort by: Most Recent |
|  | #8 | "e[pidemiology](https://www.ncbi.nlm.nih.gov/mesh/68004813)"[Mesh Terms] Sort by: Most Recent |
|  | #9 | "prevalence"[Title/Abstract] OR "incidence"[Title/Abstract] OR "e[pidemiology](https://www.ncbi.nlm.nih.gov/mesh/68004813)"[Title/Abstract] Sort by: Most Recent |
|  | #10 | #1 OR #2 Sort by: Most Recent |
|  | #11 | #3 OR #4 #5 Sort by: Most Recent |
|  | #12 | #6 OR #7 OR #8 OR #9 Sort by: Most Recent |
|  | #13 | #10 AND #11 AND #12 Sort by: Most Recent |
| Web of Science | #1 | **TS=(**[neoplasms](https://www.ncbi.nlm.nih.gov/mesh/68009369)**) OR TS=(cancer**) OR TS=(tumor) OR TS=(tumour) OR TS=(carcinoma) OR TS=(malignancy) |
|  | #2 | **TS=(sleep) OR TS=(insomnia) OR** TS=(sleep disturbances) OR **TS=(**sleep disorders) OR **TS=(**[sleep initiation and maintenance disorders](https://www.ncbi.nlm.nih.gov/mesh/68007319)) |
|  | #3 | TS=(prevalence) OR **TS=(incidence) OR TS=(**e[pidemiology](https://www.ncbi.nlm.nih.gov/mesh/68004813)) |
|  | #4 | #1 AND #2 AND #3 |
| Scopus | #1 | ABS("[neoplasms](https://www.ncbi.nlm.nih.gov/mesh/68009369)" OR "cancer" OR "tumor" OR "tumour" OR "carcinoma" OR "malignancy") |
|  | #2 | ABS("sleep" OR "insomnia" OR "sleep disturbances" OR "sleep disorders" OR "[sleep initiation and maintenance disorders](https://www.ncbi.nlm.nih.gov/mesh/68007319)") |
|  | #3 | ABS ("incidence" OR "prevalence" OR "e[pidemiology](https://www.ncbi.nlm.nih.gov/mesh/68004813)") |
|  | #4 | #1 AND #2 AND #3 |
| Embase | **#1** | **'**[neoplasms](https://www.ncbi.nlm.nih.gov/mesh/68009369)**'/exp** |
|  | **#2** | 'cancer':ti,ab,kw OR 'tumor':ti,ab,kw OR 'tumour':ti,ab,kw OR 'carcinoma':ti,ab,kw OR 'malignancy':ti,ab,kw |
|  | #3 | **'insomnia'/exp** |
|  | #4 | 'sleep**':ti,ab,kw OR 'insomnia':ti,ab,kw OR '**sleep disturbances**':ti,ab,kw OR '**sleep disorders**':ti,ab,kw OR '**[sleep initiation and maintenance disorders](https://www.ncbi.nlm.nih.gov/mesh/68007319)**':ti,ab,kw** |
|  | #5 | 'prevalence**':ti,ab,kw OR 'incidence':ti,ab,kw OR '**e[pidemiology](https://www.ncbi.nlm.nih.gov/mesh/68004813)**':ti,ab,kw** |
|  | #6 | #1 OR #2 |
|  | #7 | #3 AND #4 |
|  | #8 | #5 AND #6 AND #7 |
| PsycINFO | S1 | **TI** [neoplasms](https://www.ncbi.nlm.nih.gov/mesh/68009369) **OR TI cancer** OR TI tumor OR TI tumour OR TI carcinoma OR TI malignancy |
|  | S2 | TI sleep **OR TI insomnia OR TI** sleep disturbances **OR TI** sleep disorders **OR TI** [sleep initiation and maintenance disorders](https://www.ncbi.nlm.nih.gov/mesh/68007319) |
|  | S3 | TI prevalence **OR TI incidence OR TI** e[pidemiology](https://www.ncbi.nlm.nih.gov/mesh/68004813) |
|  | S4 | S1 AND S2 AND S3 |
| Cochrane Library | #1 | MeSH descriptor: [Neoplasms] explode all trees |
|  | #2 | (cancer):ti,ab,kw OR (tumor):ti,ab,kw OR (tumour):ti,ab,kw OR (carcinoma):ti,ab,kw OR (malignancy):ti,ab,kw |
|  | #3 | MeSH descriptor: [Sleep Initiation and Maintenance Disorders] explode all trees |
|  | #4 | (sleep)**:ti,ab,kw OR (insomina**)**:ti,ab,kw OR (**sleep disturbances)**:ti,ab,kw OR (**sleep disorders)**:ti,ab,kw** |
|  | #5 | (prevalence)**:ti,ab,kw OR (incidence**)**:ti,ab,kw OR (**e[pidemiology](https://www.ncbi.nlm.nih.gov/mesh/68004813))**:ti,ab,kw** |
|  | #6 | #1 OR #2 |
|  | #7 | #3 OR #4 |
|  | #8 | #5 AND #6 AND #7 |
| CINAHL | S1 | **TI** [neoplasms](https://www.ncbi.nlm.nih.gov/mesh/68009369) **OR TI cancer** OR TI tumor OR TI tumour OR TI carcinoma OR TI malignancy |
|  | S2 | TI sleep **OR TI insomnia OR TI** sleep disturbances **OR TI** sleep disorders **OR TI** [sleep initiation and maintenance disorders](https://www.ncbi.nlm.nih.gov/mesh/68007319) |
|  | S3 | TI prevalence **OR TI incidence OR TI** e[pidemiology](https://www.ncbi.nlm.nih.gov/mesh/68004813) |
|  | S4 | S1 AND S2 AND S3 |
| CNKI |  | (SU="肿瘤"+ "恶性肿瘤" + "癌症") AND (SU="睡眠" + "失眠"+ "睡眠障碍") AND (SU="发生率" + "患病率") |
| Wanfang |  | 题名或关键词:(肿瘤 OR 恶性肿瘤 OR 癌症) and 题名或关键词:(睡眠 OR 失眠 OR 睡眠障碍) and 题名或关键词:(发生率 OR 患病率) |
| VIP |  | (题名或关键词=肿瘤 OR 恶性肿瘤OR 癌症) and (题名或关键词=睡眠 OR 失眠 OR 睡眠障碍) and (摘要=发生率 OR 患病率) |
| CBM |  | ("肿瘤"[标题] OR "恶性肿瘤"[标题] OR "癌症"[标题] ) AND ( "睡眠"[标题] OR "失眠"[标题] OR "睡眠障碍"[标题]) |

Table S2. Quality assessment results of included studies

| Study | Q1 | Q2 | Q3 | Q4 | Q5 | Q6 | Q7 | Q8 | Q9 | Overall |
| --- | --- | --- | --- | --- | --- | --- | --- | --- | --- | --- |
| Aggeli et al. (2021) | N | N | N | Y | Y | Y | Y | Y | Y | L |
| Amani et al. (2024) | N | N | U | Y | Y | Y | Y | Y | U | M |
| Chen et al. (2021) | N | U | U | Y | Y | Y | Y | Y | U | M |
| Cho et al. (2023) | N | N | U | Y | Y | Y | Y | Y | Y | L |
| Desai et al. (2013) | N | U | U | Y | Y | Y | Y | Y | Y | L |
| Desautels et al. (2017) | Y | Y | U | Y | Y | Y | Y | Y | Y | L |
| Dirksen et al. (2019) | N | N | N | Y | Y | Y | Y | Y | U | M |
| Eslamijouybari et al. (2025) | Y | N | Y | Y | Y | Y | Y | Y | Y | L |
| Fleming et al. (2019) | N | N | U | Y | Y | Y | Y | Y | N | M |
| Galvin et al. (2022) | Y | N | U | Y | Y | Y | Y | Y | N | L |
| Geng et al. 2023 | N | N | N | U | Y | Y | Y | Y | Y | M |
| Hajj et al. (2021) | N | N | U | Y | Y | Y | Y | Y | U | M |
| Haque et al. (2021) | Y | Y | U | Y | Y | Y | Y | Y | Y | L |
| He et al. (2022) | N | N | N | Y | Y | Y | Y | Y | Y | L |
| Hinz et al. (2025) | Y | U | Y | Y | U | Y | Y | Y | U | L |
| Hoang et al. (2020) | N | N | U | Y | Y | Y | Y | Y | U | M |
| Huang et al. (2021) | N | U | Y | Y | Y | Y | Y | Y | Y | L |
| Li et al. (2021) | Y | Y | N | N | Y | Y | Y | Y | U | L |
| Li et al. (2024) | Y | N | U | Y | Y | Y | Y | Y | Y | L |
| Liao et al. (2023) | Y | Y | U | Y | Y | Y | Y | Y | U | L |
| Liou et al. (2023) | Y | U | Y | Y | Y | Y | Y | Y | Y | L |
| Liu et al. (2023) | N | N | Y | Y | Y | Y | Y | Y | Y | L |
| Madkhali et al. (2022) | Y | N | Y | Y | Y | Y | Y | Y | N | L |
| Mercadante et al. (2015) | Y | N | Y | Y | Y | Y | Y | Y | Y | L |
| Nishiura et al. (2015) | N | N | N | Y | Y | Y | Y | Y | U | M |
| Noyan et al. (2023) | N | N | N | Y | Y | Y | Y | Y | U | M |
| Pang et al. (2022) | Y | N | Y | Y | Y | Y | Y | Y | Y | L |
| Price et al. (2009) | Y | N | Y | Y | Y | Y | Y | Y | U | L |
| Ruiz-Marin et al. (2021) | N | N | N | Y | Y | Y | Y | Y | U | M |
| Savard et al. (2005) | N | N | U | Y | Y | Y | Y | Y | N | M |
| Sun et al. (2011) | Y | N | N | Y | Y | Y | Y | Y | Y | L |
| Sun et al. (2020) | Y | N | Y | Y | Y | Y | Y | Y | Y | L |
| Taskaynatan et al. (2025) | Y | N | U | Y | Y | Y | Y | Y | Y | L |
| Trendowski et al. (2025) | Y | N | Y | Y | Y | Y | Y | Y | Y | L |
| Ueno et al. (2022) | Y | Y | Y | Y | Y | Y | Y | Y | U | L |
| Wang et al. (2022) | N | N | U | Y | Y | Y | Y | Y | U | M |
| Yeo et al. (2021) | N | N | U | Y | Y | Y | Y | Y | U | M |
| Yi et al. (2022) | N | N | U | Y | Y | Y | Y | Y | Y | M |
| Zhang et al. (2024) | Y | Y | Y | Y | Y | Y | Y | Y | Y | L |

Notes: Y: Yes, N: No, U: Unclear. L: Low risk, M: Moderate risk, H: High risk.

Q1: Was the sample frame appropriate to address the target population?

Q2: Were study participants sampled in an appropriate way?

Q3: Was the sample size adequate?

Q4: Were the study subjects and the setting described in detail?

Q5: Was the data analysis conducted with sufficient coverage of the identified sample?

Q6: Were valid methods used for the identification of the condition?

Q7: Was the condition measured in a standard, reliable way for all participants?

Q8: Was there appropriate statistical analysis?

Q9: Was the response rate adequate, and if not, was the low response rate managed appropriately?

Each study was categorized by risk of bias as follows: high risk if ≤ 49% of items are responded to with "yes;" moderate risk if 50%-69% of items are responded to with "yes; "and low risk if ≥ 70% of items are responded to with "yes."

Figure S1. The pooled prevalence of clinically significant insomnia among cancer patients based on country income level.

Figure S2. The pooled prevalence of clinically significant insomnia among cancer patients based on country study design.

Figure S3. The pooled prevalence of clinically significant insomnia among cancer patients based on cancer category.

Figure S4. The pooled prevalence of clinically significant insomnia among cancer patients based on assessment tool.

Table S3 Meta-regression results

| Variables | B | 95%CI | t | p | Adjusted R^2^ |
| --- | --- | --- | --- | --- | --- |
| Publication year | 0.0044542 | -0.012003, 0.0209113 | 0.55 | 0.587 | -1.98% |
| Sample size | -0.0002653 | -0.0005112, -0.0000194 | -2.19 | 0.035 | 9.05% |
| Mean age (years) | 0.0000176 | -0.0109638, 0.111071 | 0.01 | 0.989 | -3.86% |
| Proportion of female participants | -0.0096058 | -0.0007591, 0.0199708 | 1.88 | 0.068 | 6.68% |

Fig. S5 Egger's test results

Fig. S6 Trim-fill analysis results

Table S4 The results of the sensitivity analysis

| **Study omitted** | **Prevalence (%)** | **95% CI** |
| --- | --- | --- |
| Aggeli et al. (2021) | 34.1 | (27.5, 40.8) |
| Cha et al. (2017) | 34.4 | (27.6, 41.1) |
| Chen et al. (2021) | 35.2 | (28.3, 42.1) |
| Cho et al. (2023) | 33.6 | (27.0, 40.2) |
| Desai et al. (2013) | 35 | (28.2, 41.9) |
| Desautels et al. (2017) | 35.4 | (28.5, 42.2) |
| Dirksen et al. (2019) | 34.2 | (27.5, 40.9) |
| Eslamijouybari et al. (2025) | 35.1 | (28.2, 41.9) |
| Fleming et al. (2019) | 33.4 | (26.9, 39.9) |
| Galvin et al. (2022) | 35 | (28.2, 41.7) |
| Geng et al. (2023) | 33.7 | (27.0, 40.3) |
| Hajj et al. (2021) | 34.8 | (28.1, 41.6) |
| Haque et al. (2021) | 35.4 | (28.6, 42.1) |
| He et al. (2022) | 35.2 | (28.5, 42.0) |
| Hinz et al. (2025) | 34.2 | (27.5, 40.9) |
| Hoang et al. (2020) | 34.4 | (27.7, 41.2) |
| Huang et al. (2021) | 34.9 | (28.1, 41.6) |
| Li et al. (2021) | 35.1 | (28.3, 42.0) |
| Li et al. (2024) | 34.6 | (27.8, 41.3) |
| Liao et al. (2023) | 34.5 | (27.8, 41.2) |
| Liou et al. (2019) | 35.1 | (28.1, 42.0) |
| Liu et al. (2023) | 34.9 | (28.1, 41.6) |
| Madkhali et al. (2022) | 33.7 | (27.2, 40.3) |
| Mercadante et al. (2015) | 33.3 | (27.9, 38.7) |
| Nishiura et al. (2015) | 34.7 | (28.0, 41.4) |
| Noyan et al. (2023) | 35.2 | (28.4, 41.9) |
| Pang et al. (2022) | 35.3 | (28.5, 42.2) |
| Price et al. (2009) | 35.1 | (28.2, 42.0) |
| Ruiz-Marin et al. (2021) | 34.9 | (28.2, 41.6) |
| Savard et al. (2005) | 35.1 | (28.3, 41.9) |
| Sun et al. (2011) | 34.9 | (28.2, 41.7) |
| Sun et al. (2020) | 34.5 | (27.8, 41.3) |
| Taskaynatan et al. (2025) | 35.1 | (28.3, 41.9) |
| Trendowski et al. (2025) | 35.3 | (28.4, 42.2) |
| Ueno et al. (2022) | 34.6 | (27.8, 41.4) |
| Wang et al. (2022) | 34.0 | (27.3, 40.6) |
| Yeo et al. (2021) | 34.1 | (27.4, 40.7) |
| Yi et al. (2022) | 33.8 | (27.2, 40.4) |
| Zhang et al. (2024) | 35.2 | (28.4, 42.1) |
